# Band gap engineering in lead free halide cubic perovskites GaGeX_3_ (X = Cl, Br, and I) based on first-principles calculations

**DOI:** 10.1039/d4ra00224e

**Published:** 2024-03-25

**Authors:** Md. Amran Sarker, Md Mehedi Hasan, Md. Al Momin, Ahmad Irfan, Md. Rasidul Islam, Ahmed Sharif

**Affiliations:** a Department of Materials and Metallurgical Engineering, Bangladesh University of Engineering & Technology (BUET) Dhaka Bangladesh asharif@mme.buet.ac.bd; b Department of Materials Science and Engineering, Khulna University of Engineering & Technology (KUET) Khulna 9203 Bangladesh; c Department of Chemistry, College of Science, King Khalid University PO. Box 9004 Abha 61413 Saudi Arabia; d Department of Electrical and Electronic Engineering, Bangamata Sheikh Fojilatunnesa Mujib Science & Technology University Jamalpur 2012 Bangladesh

## Abstract

Lead-free inorganic Ge-based perovskites GaGeX_3_ (X = Cl, Br, and I) are promising candidates for solar cell applications due to their structural, mechanical, electrical, and optical properties. In this work, we performed density functional theory (DFT) calculations using the CASTEP module to investigate these properties in detail. We found that the lattice parameters and cell volumes increase with the size of the halogen atoms, and that all the compounds are stable and ductile. GaGeBr_3_ has the highest ductility, machinability, and lowest hardness, while GaGeCl_3_ has the highest anisotropy. The band gap values, calculated using the GGA-PBE and HSE06 functionals, show a direct band gap at the R–R point, ranging from 0.779 eV and 1.632 eV for GaGeCl_3_ to 0.330 eV and 1.140 eV for GaGeI_3_. The optical properties, such as absorption coefficient, conductivity, reflectivity, refractive index, extinction coefficient, and dielectric function, are also computed and discussed. We observed that the optical properties improve with the redshift of the band gap as Cl is replaced by Br and I. GaGeI_3_ has the highest dielectric constant, indicating the lowest recombination rate of electron–hole pairs. Our results suggest that GaGeX_3_ (X = Cl, Br, and I) can be used as effective and non-toxic materials for multijunction solar cells and other semiconductor devices.

## Abstract 2

Lead-free inorganic Ge-based perovskites GaGeX_3_ (X = Cl, Br, and I) are promising candidates for solar cell applications due to their structural, mechanical, electrical, and optical properties.

## Graphical abstract

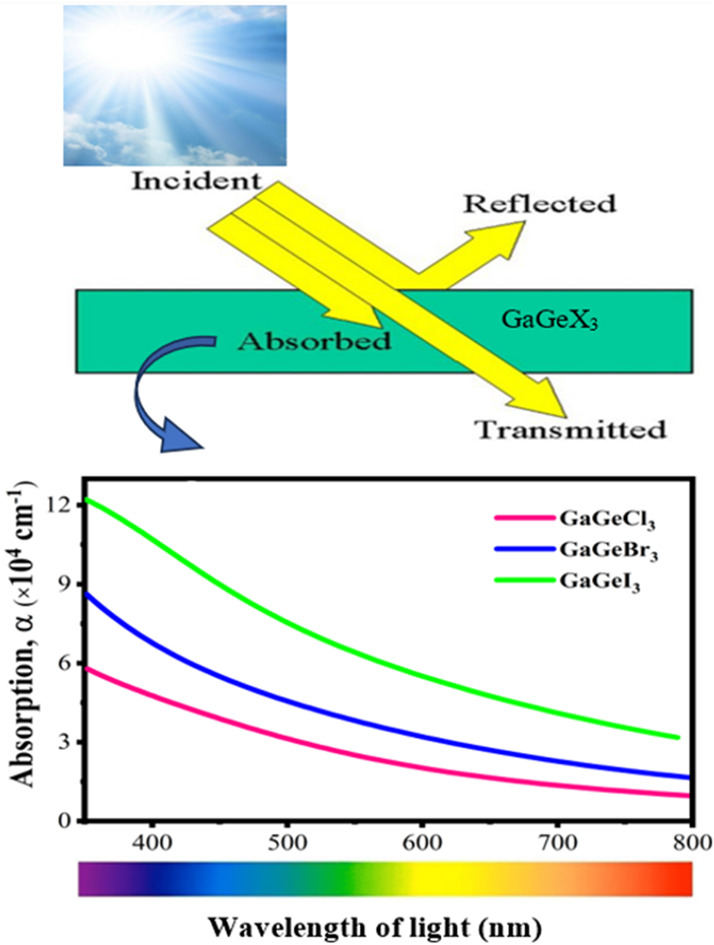

## 1. Introduction

The need for energy increases along with the world's population. It's no exaggeration to argue that inexpensive, abundant energy is essential to the operation of our modern, industrialized society. The majority of the energy used around the globe is non-renewable, such as coal, gas, oil, *etc.* Unfortunately, with time, nonrenewable energy sources will run out. The majority of the world's energy output comes from burning fossil fuels, which releases greenhouse gases that contribute to climate change. This is one of the main drawbacks of using non-renewable energy. The most important decision for the environment may be to rely on renewable energy sources, such as solar energy, as doing so would help reduce greenhouse gas emissions to a tolerable level and lessen the negative consequences of global warming. Because of the capability to convert light into electricity in an unconventional manner,^1–8^ modern solar cell technologies are widely recognized in both practical engineering and fundamental scientific studies^9–15^ as a means of producing clean, renewable power. In its most typical configuration, a solar cell consists of three layers. The layers are as follows: a hole transport layer at the bottom, an absorber layer above, and an electron transport layer below. Because of the creation of electron–hole pairs and the absorption of electromagnetic radiation, the absorbent layer is indispensable. The geometrical, optical, and electrical characteristics of the three layers above have a significant impact on the efficiency of light-generated currents and the configuration of the core parts.

Perovskite compounds (ABX_3_) have been considered reliable options for light-absorbing materials for renewable energy supply throughout the past ten years due to their progressively increasing power conversion efficiency (PCE) and lower production cost.^16–18^ In recent years, perovskite solar cells (MAPbI_3_ where MA = CH_3_NH_3_) have achieved record highs in PCE >25%.^19,20^ However, the compound becomes unstable in various kinds of environmental circumstances due to the presence of organic molecules (MA), which narrows the range of potential uses.^21,22^ Furthermore, the toxicity of the heavy hazardous element Pb endangers the environment and hinders its extensive commercialization.^23–25^ Substituting inorganic alkali cations (Cs^+^, Rb^+^) for the organic ones may stabilize the system^26^ while replacing lead with a non-toxic member of group 14, like Sn or Ge, can get rid of the toxicity.^27,28^ Lead-free germanium-based lead-free perovskite solar cells synthesized with CsGeX_3_ (X = halogen) have been reported to have a power conversion efficiency (PCE) of about 4.92%.^29,30^ However, doping it with tin (CsSn0.5Ge0.5I3) caused an increase of 7.11%. Furthermore, PCEs higher than 10% have been attained using CsSnI3-based perovskite solar cells.^31^

The previous discussion indicates that organic-inorganic halide perovskites (MAPbI_3_) have a higher PCE than Pb-free perovskite solar cells. Thus, to create high PCE (%) absorber layers for solar cells, researchers are always looking for novel Pb-free perovskite materials. The factors that affect power conversion efficiency (PCE) are bandgap, stability, high carrier mobility, low excitation binding energy, and high absorption. Therefore, more research is needed to develop inorganic lead-free perovskite solar cells. However, a lot of study has been done on Ge-based halide perovskites, much like other perovskite materials. Furthermore, GaGeX_3_ (X = Cl, Br, and I) is a non-toxic material with promising physical features that have not yet been studied.

In this study, we investigate the properties of inexpensive and non-toxic lead-free gallium (Ga) based cubic halide perovskite GaGeX_3_ (X = Cl, Br, and I), which have been reported to exhibit high stability and can be applicable in multijunction solar cells. We use density functional theory (DFT) calculations to study the lattice parameter, mechanical stability, thermodynamically stability, structural stability, ductile and brittle behavior, anisotropy, bandstructure, density of states and optical properties (complex dielectric function and refractive index, absorption coefficient, conductivity, reflectivity and loss function) of these compounds. We aim to understand the effects of the halogen atoms on the properties of GaGeX_3_ and to identify the most suitable compound for photovoltaic applications. This research may offer a way to discover an effective and lead-free photovoltaic material that can overcome the limitations of the lead-based perovskites.

## 2. Computational methodology

**Fig. 1:**
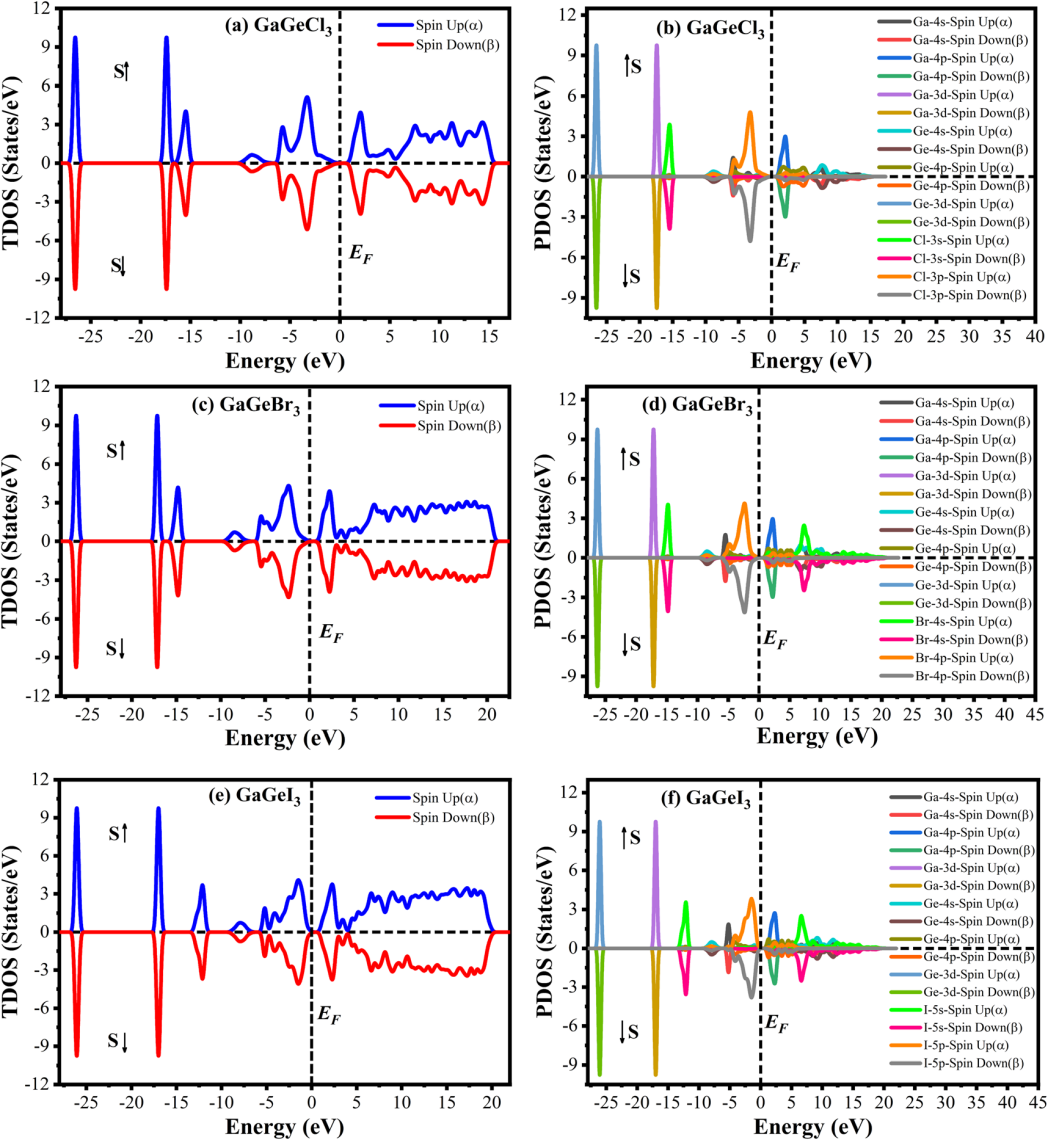
Density of state of spin up and spin down of GaGeX_3_ (X = Cl, Br, and I). [a, c and e] indicate total density of state (TDOS) and [b, d and f] indicate partial density of state (PDOS) calculated by using spin polarized DFT + U.

The Cambridge Serial Total Energy Package (CASTEP)^32^ has been used to do the first-principles calculations inside the framework of DFT.^33,34^ CASTEP mainly uses the plane-wave pseudopotential total energy technique to study the physical features.^35^ The general gradient approximation (GGA) using the Perdew–Berke–Emzerhof (PBE) functional is used to calculate the electronic exchange-correlation energy.^36^ The Vanderbilt-type ultrasoft pseudopotential is intended to exist to regulate the electron–ion interactions.^37^ The optimal crystal structure has been assured by using the Broyden–Fletcher–Goldfarb–Shanno (BFGS) approach.^38^ The plane wave energy cutoff was set at 600 eV with *k*-points 7 × 7 × 7 to achieve the optimal structure and the characteristics calculations. The *k*-point's Brillouin zone sampling was done using the Monkhorst–Pack approach.^39^ We performed spin-polarized DFT + U calculations to determine whether GaGeX_3_ (where X = Cl, Br, and I) exhibits magnetic properties. It was considered that the value of U for both Ga and Ge is 4 eV. Since the total density of states for spin up and spin down electrons is symmetrical, as depicted in Fig. 1, there is no net magnetic moment. The cancelation of magnetic moments between spins leads to this conclusion. Hence, GaGeX_3_ (where X = Cl, Br, and I) is considered non-magnetic. Consequently, we set the spin polarization to be non-polarized in all calculations, also known as ‘spin-restricted’ calculations, where the same orbitals are used for both alpha and beta spins. Higher *k*-points are employed in the optical property calculation process. To calculate the bandgap more accurately, we used the HSE06 functional with Quantum ESPRESSO. Under typical conditions, the stress–strain technique has been used to derive the elastic stiffness constants of the studied model.^40^ The strain magnitude was set at 0.003 in order to attain the optimal results. The following levels of convergence were established: 5 × 10^−6^ eV per atom for total energy; 5 × 10^−4^ Å for maximum displacements; 0.01 eV Å^−1^ for maximum force; and 0.02 GPa for maximum stress.

## 3. Results & discussion

### 3.1 Structural properties

**Fig. 2:**
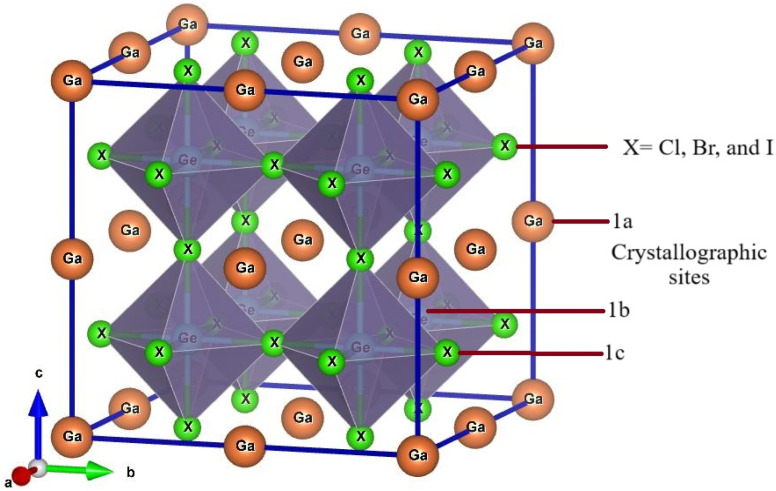
Optimized crystal structure of GaGeX_3_ (X = Cl, Br, and I).

Ge-based halide perovskite GaGeX_3_(X = Cl, Br, and I), belongs to space group *Pm*3̄*m* (#221) and crystallizes as a cubic structure. The VESTA software illustrates the crystal structure of GaGeX_3_, which is displayed in Fig. 2. GaGeX_3_ is a crystal lattice composed of five atoms, where X represents the halogens (Cl, Br, and I). Wyckoff position 1a is occupied by the Ga atom, which is positioned at coordinates *A* (0, 0, 0), while Wyckoff position 1b is occupied by the Ge atom, which is placed at coordinates *B* (0.5, 0.5, 0.5). Wyckoff position 3c is occupied by the X (X = Cl, Br, and I) atoms at positions X (0.5, 0.5, 0). The desire for site occupation in ABX_3_ perovskite materials depends on several variables, including ionic radii, electronegativity, and crystallization energy.^41^ GaGeCl_3_ is likely to be more stable than GeGaCl_3_ because of the better size fit between Ga and Cl and the negligible effect of electronegativity differences. Recently some research papers have been published on GaGeF_3_, where aurhors have proved that the preference site of Ga is *A* (0, 0, 0) site.^42,43^ The Simulated structural parameters are tabulated in Table 1 along with formation energy and band gap. For GaGeCl_3_, our computed lattice constant is 5.220 Å. However, when the atomic number of halides (X) increases, the lattice parameter also rises and it becomes 5.482 Å, and 5.854 Å for GaGeBr_3_, and GaGeI_3_ respectively. Due to the atomic size is proportional to the atomic number. Similar to the lattice parameter, cell volume also increases with the atomic number of halogens (X), and these are 142.277 Å^3^, 164.729 Å^3^, and 200.568 Å^3^ respectively for GaGeCl_3_, GaGeBr_3_, and GaGeI_3_.

**Table 1:** The Computed lattice constants (*a*), cell volume (*V*), formation energy(Δ*E*_f_), final enthalpy *E*_total_ (GaGeX_3_), and energy gap (*E*_g_) of GaGeX_3_ (X = Cl, Br, and I)

Compound	*a* (Å)	*V* (Å^3^)	Δ*E*_f_ (eV per atom)	*E*_total_ (GaGeX_3_) (eV)	*E*_g_ (eV) (PBE)	*E*_g_ (eV) (HSE06)
GaGeCl_3_	5.220	142.277	−3.434	−5.9568 × 10^3^	0.779	1.632
GaGeBr_3_	5.482	164.729	−3.106	−6.0788 × 10^3^	0.462	1.284
GaGeI_3_	5.854	200.568	−2.750	−7.0753 × 10^3^	0.330	1.140

During the synthesis of a material, phase separation can occur, which is a general phenomenon. For example, GaX and GeX_2_ phases can separate during synthesis of GaGeX_3_. The stability of GaGeX_3_ is confirmed by the Born stability criteria, the formation energy, final enthalpy and phonon analysis. The Born stability calculation is discussed briefly in the mechanical properties section. The compounds are more stable when their final enthalpy is negative since it shows that their enthalpy of formation is less than that of the reactants. Formation energy can be used to predict the phase stability and the tendency of a compound to decompose. A negative formation energy means that the compound has a lower energy than its constituent elements, and therefore it is more stable.^44,45^ Since all of our interested compounds have high negative values of formation energy and final enthalpy, it is considered that there is no phase separation during the synthesis of those compounds.

Formation energy (Δ*E*_f_) is one of the most significant variables in estimating the crystal stability of a structure. The formation energy of GaGeX_3_ (X = Cl, Br, and I) is calculated by using the following formula, and tabulated in Table 1:^46^
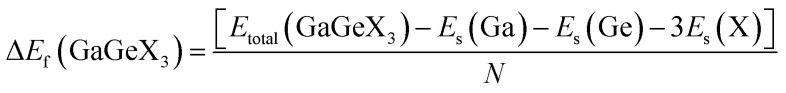
Here, *E*_total_ (GaGeX_3_) indicates the total energy of the unit cell, whereas *E*_s_ (Ga), *E*_s_ (Ge), and *E*_s_ (X) refer to the energy of Cs, Cd, and X (Cl, Br, and I). Furthermore, the negative values of Δ*E*_f_ (GaGeX_3_) validate the thermodynamic stability of GaGeX_3_ (X = Cl, Br, and I).^46^

**Fig. 3:**
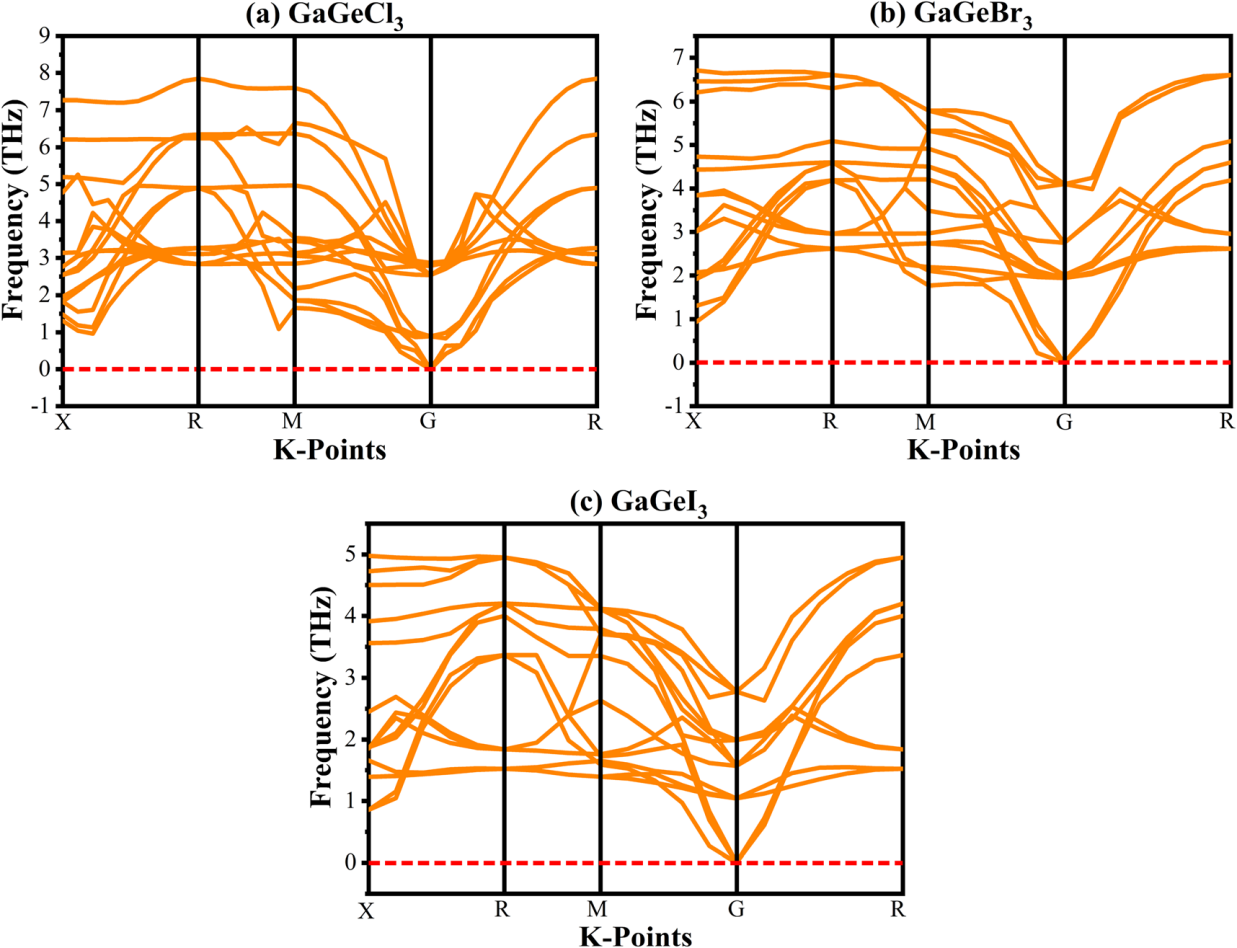
Phonon dispersion curve of (a) GaGeCl_3_, (b) GaGeBr_3_, and (c) GaGeI_3_.

Analyzing the phonon dispersion curve is crucial for assessing dynamic stability in crystalline materials. These curves depict the relationship between the frequency of lattice vibrations (phonons) and their corresponding wave vectors. The phonon dispersion relation describes how the phonon frequency varies with wave vector. Phonons are essential for determining thermal conductivity, specific heat, and mechanical stability of materials. Negative frequencies in the dispersion curve indicate instability. If a material exhibits negative frequencies, it implies that certain vibrational modes are energetically unfavorable, leading to potential lattice distortions or even collapse. The phonon dispersion curves for GaGeX_3_ were analyzed and visualized in Fig. 3. It indicates that all vibrational modes within GaGeX_3_ are energetically stable as there are no negative frequencies. Based on this analysis, it can be concluded confidently that GaGeX_3_ is dynamically stable. Its crystal lattice remains intact, and there are no indications of instability.

### 3.2 Mechanical properties

Material's elastic and mechanical features are essential for their implementation in devices. Elastic constants are very crucial for predicting the physical properties of a crystal under equilibrium circumstances. This helps determine the solid's hardness, ductile-brittle behaviour, bonding nature, and other properties, as well as the solid's mechanical stability.^47,48^Table 2 lists the elastic constants of GaGeX_3_ (X = Cl, Br, and I), which are calculated using the “stress–strain” principle. To evaluate the mechanical stability of a cubic structure, the well-known Born stability criteria are utilized,^49^ which is *C*_11_ > 0, *C*_11_ + 2*C*_12_ > 0, *C*_11_ − *C*_12_ > 0, and *C*_44_ > 0. To maintain mechanical stability, this equation states that *C*_11_, *C*_12_, and *C*_44_ must be larger than zero. Our simulated elastic constants which are tabulated in Table 2 confirm that GaGeX_3_ (X = Cl, Br, and I) is mechanically stable. Additionally, greater incompressibility in the *x*-direction is indicated by a larger value of *C*_11_. Among these three minerals, GaGeCl_3_ has the greatest value of *C*_11_, as Table 2 demonstrates. Therefore, in the *x*-direction, GaGeCl_3_ has the maximum incompressibility. Moreover, The stiffness and resistance to deformation of the crystal are indicated by greater values of the elastic constants (*C*_11_, *C*_12_, and *C*_44_). Given those three compounds, GaGeCl_3_ exhibits the best degree of stiffness and deformation resistance. Normally, elastic constant *C*_12_ along the (100) plane depends on the atomic arrangement in the crystal lattice as well as the interatomic forces. Elastic constants are often lower at larger interatomic distances since the intensity of interatomic forces decreases with such distance. Here we see when halogen Cl is replaced by Br, the elastic constant *C*_12_ is higher compared to Cl-containing material. This contradiction may occur because of its crystal structure and bonding properties, GaGeBr_3_ may be more resistant to shear deformation along the (100) plane than GaGeCl_3_, even though it has larger interatomic distances. And because of increasing elastic constant of *C*_12_ for GaGeBr_3_ that changing the other mechanical properties those are related to *C*_12_ such as Bulk modulus (*B*), Pugh's ratio (*B*/*G*), Poisson's ratio (*ν*), Hardness (*H*_v_), and Machinability index (*μ*_M_).

**Table 2:** The calculated elastic constants, *C*_*ij*_ (in GPa), Cauchy pressure, *C*_12_–*C*_44_, bulk modulus, *B* (in GPa), shear modulus, *G* (in GPa), Young's modulus, *Y* (in GPa), Pugh's ratio (*B*/*G*), Poisson's ratio, *ν*, Vickers hardness, *H*_v_, and machinability index, *μ*_M_ of GaGeX_3_ (X = Cl, Br, and I) compounds

Compound	*C*_11_	*C*_12_	*C*_44_	*C*_12_–*C*_44_	*B* (GPa)	*G* (GPa)	*Y*	*B*/*G*	*υ*	*H*_v_	*μ*_M_
GaGeCl_3_	59.76	11.38	6.24	5.14	27.51	11.15	29.46	2.47	0.32	1.82	4.41
GaGeBr_3_	58.64	15.59	5.61	9.97	29.94	9.98	26.93	3.00	0.35	1.34	5.33
GaGeI_3_	43.88	6.24	5.53	0.71	18.79	9.27	23.89	2.03	0.29	1.99	3.40

The Vigot–Reuss–Hill (VRH) assumption may be utilized to compute a solid's mechanical behaviour by applying elastic constants. Young's modulus (*Y*), shear modulus (*G*), and bulk modulus (*B*) can be used to express resistance to longitudinal, shear, and volume deformation, respectively. We computed *Y*, *G*, and *B* of GaGeX_3_ (X = Cl, Br, and I) in this work, and Table 2 lists them.
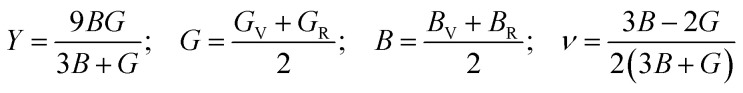


To determine the ductile-brittle nature of a material Poission's ratio(*ν*), Pugh's ratio (*B*/*G*), and Cauchy pressure *C*_12_–*C*_44_is utilized.^50^ Regarding any material, the critical values of *ν* and *B*/*G* are respectively 0.26 and 1.75. The ductile character of materials is demonstrated by values larger than 1.75 and 0.26 for Pugh's and Poission's ratios, respectively. Moreover, as the value increases the degree of ductility also increases. Table 2 elucidates that, our interested all three materials are ductile, but GaGeBr_3_ exhibits the highest degree of ductility as it shows the highest value of Pugh's and Poission's ratio, whilst GaGeI_3_ exhibits minimal ductility among them because of the lowest value of Pugh's and Poission's ratio. Similar to *ν*, and *B*/*G*, Cauchy pressure is also a crucial parameter to identify the ductile-brittle nature of a material. The brittle character of the material is shown by the negative Cauchy pressure (*C*_12_–*C*_44_) value, whereas a positive reading of Cauchy pressure reflects its ductile behaviour.^51^Table 2 elicits that the Cauchy pressure of GaGeX_3_ (X = Cl, Br, and I) is positive, which reconfirms that all three materials are ductile, and GaGeBr_3_ is the most ductile material because of the highest value of Cauchy pressure.

A material's capacity to withstand plastic deformation is defined by its hardness. The following formula is used to calculate hardness:^52^
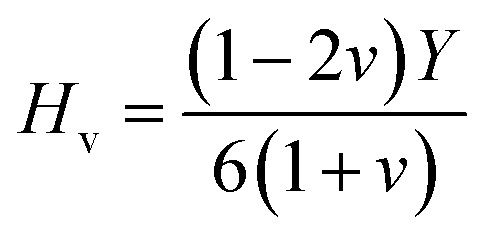


As seen in Table 2, GaGeI_3_ has a hardness of 1.99, which is the maximum among all three materials, and when I is replaced by Cl and Br, hardness is decreased by about 1.09 and 1.49 respectively. The manufacturing sector is significantly impacted by the machinability index *μ*_M_, which determined a substance's cutting power, the most efficient way to use a machine and plastic strain. Table 2 shows that, The *μ*_M_ value of GaGeCl_3_ is 4.41 which is 1.21 times lower than GaGeBr_3_ and 1.30 times higher than GaGeI_3_. GaGeBr_3_ exhibits the highest *μ*_M_, which states that it is considerably more lubricating, has less friction, and has maximum machinability among all three materials, which significantly affects the production process. The following formula can be utilized to determine the machinability index:^53^
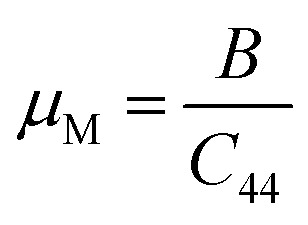


Understanding the microscopic behaviour in single and multi-crystalline materials requires an understanding of elastic anisotropy. Anisotropy in a material was primarily driven by *C*_11_. Therefore, it is crucial to look for the directionality of the elastic tensor. By studying elastic anisotropy, mechanical resilience and adaptability of solid materials can be determined under stress. The mathematical representation of elastic anisotropy is:
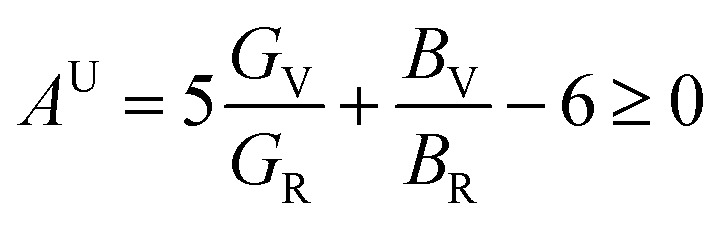


A material's isotropic state may be determined by studying its universal anisotropy (*A*^U^) value. The extent of anisotropy is expressed by the departure from the value of *A*^U^, which indicates isotropic material if it is zero. From Table 3. We observe that GaGeCl_3_ has the highest degree of anisotropy among our studied materials, as it deviates most from zero. The degree of anisotropy's order:GaGeCl_3_ > GaGeBr_3_ > GaGeI_3_

**Table 3:** Simulated shear anisotropic factors *A*_i_ (*i* = 1–3), Zener's anisotropy index (*A*), anisotropy in shear (*A*_G_), anisotropy in bulk modulus (*A*_B_), universal anisotropy index (*A*^U^), and equivalent Zener anisotropy (*A*^eq.^) of GaGeX_3_ (X = Cl, Br, and I) compounds

Compound	*A*_1_	*A*_2_	*A*_3_	*A*	*A*_G_	*A*_B_	*A* ^U^	*A* ^eq.^
GaGeCl_3_	0.2580	0.2580	0.2580	0.2580	0.4077	0	2.5606	3.8758
GaGeBr_3_	0.2608	0.2608	0.2608	0.2608	0.4017	0	2.5137	3.8339
GaGeI_3_	0.2936	0.2936	0.2936	0.2936	0.3388	0	2.0393	3.4058

The following formulas are used to calculate the percentage of anisotropy under shear (*A*_G_) and in bulk (*A*_B_) condition:
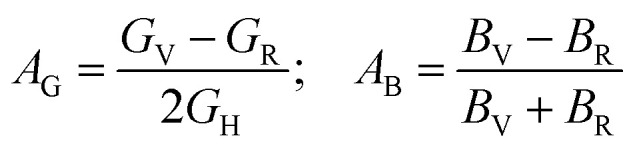
Here, when *A*_G_ and *A*_B_ are zero, the materials exhibit isotropic behaviour. Table 3 demonstrates that *A*_B_ is zero for all three compounds, and reveals that GaGeX_3_ (X = Cl, Br, and I) is isotropic under compression. However, *A*_G_ shows a deviation from zero for all studied compounds, indicating that GaGeX_3_ (X = Cl, Br, and I) exhibits anisotropic behaviour when subjected to shear, and GaGeCl_3_ shows the greatest anisotropy as it deviates most from zero among them. The shear anisotropic components *A*_i_ are evaluated using the following formula to analyze more precisely the shear anisotropy in various planes *i* = 1–3, *i.e.*, *A*_1_, *A*_2_, and *A*_3_.^54,55^

For (100) planes,
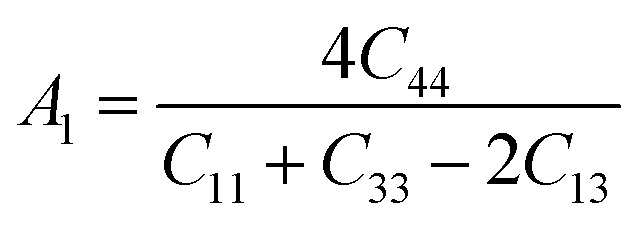
For (010) planes,
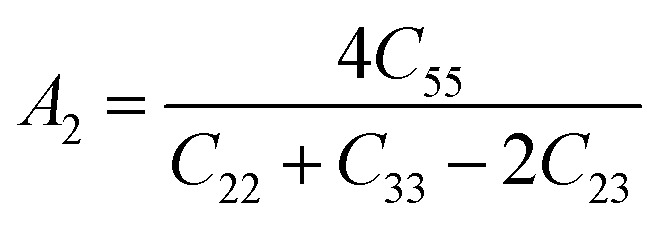


For (001) planes,
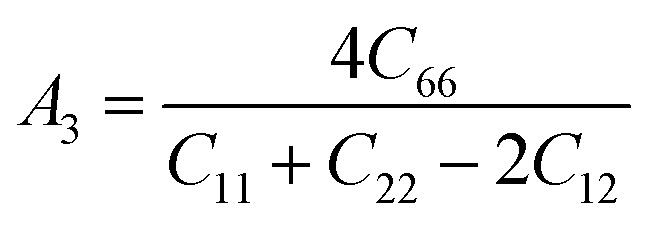


To acquire precise anisotropy, we need to determine the Zener anisotropy index (*A*) and the equivalent Zener anisotropy (*A*^eq.^). They can be evaluated by using the following formula:^56^
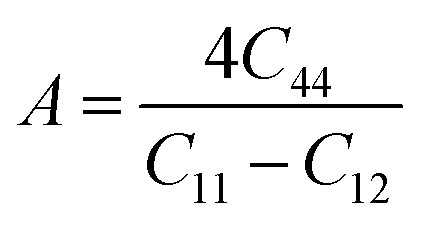

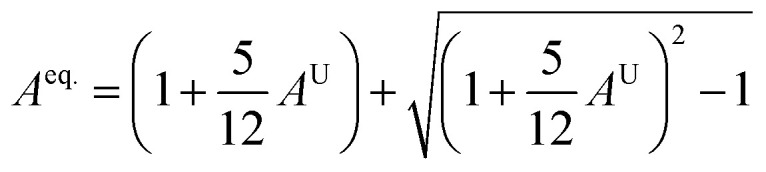


In cubic structure, *A*_1_ = *A*_2_ = *A*_3_ = *A*, and if the value of *A* = 1, it shows isotropic nature. Additionally, the degree of anisotropy is indicated by the deviation from this value. Table 3 demonstrates that all three materials are anisotropic, with GaGeCl_3_ showing the highest deviation, signifying the most pronounced anisotropic behaviour. This nature is further confirmed the *A*^eq.^. GaGeCl_3_ possesses the highest value of *A*^eq.^, which affirms that it exhibits the most anisotropic behaviour compared to the other materials.

**Fig. 4:**
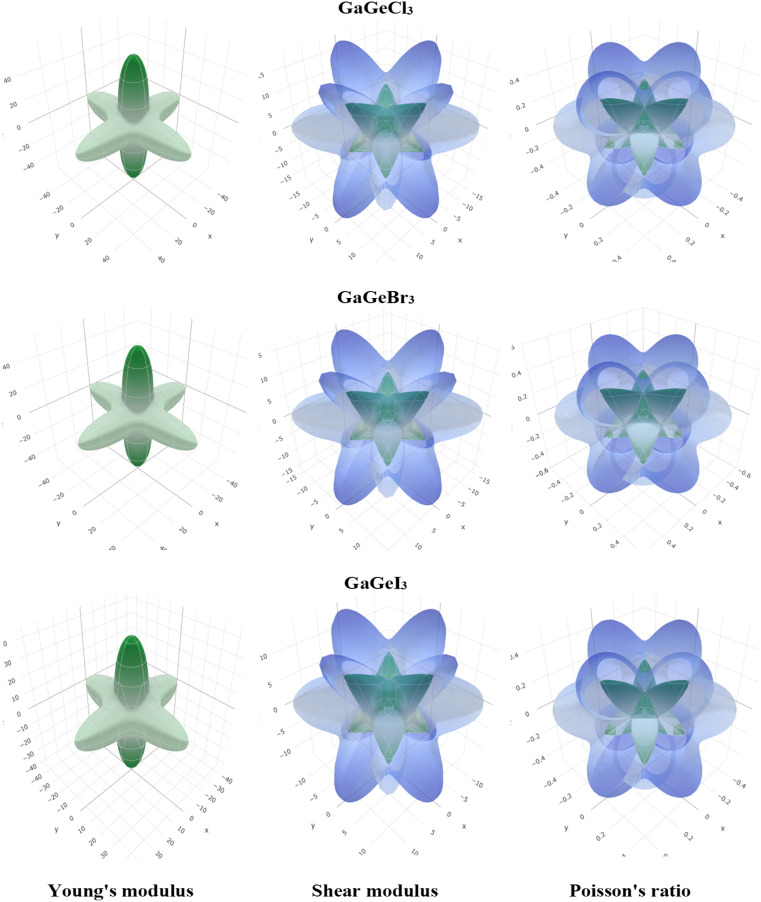
3D anisotropy contour plots of young modulus (*E*), shear modulus (*G*), and Poisson's ratio for GaGeX_3_ (X = Cl, Br and I) compounds respectively.

To visualize the anisotropy, a three-dimensional (3D) contour plot of Young modulus (*E*), shear modulus (*G*), and Poisson's ratio are shown in Fig. 4. For isotropic material, a 3D contour plot should be a spherical shape. Higher deviation from the spherical shape represents a higher degree of anisotropy. From Fig. 4, we can find a high deviation from the spherical shape of Young's modulus (*E*), shear modulus (*G*), and Poisson's ratio for GaGeX_3_ (Cl, Br, and I). For GaGeCl_3_, deviation from spherical shape is the highest among those three compounds, which proves that GaGeCl_3_ provides the highest anisotropic nature and GaGeI_3_ shows the lowest anisotropic nature among those three compounds.

### 3.3 Electronic properties

**Fig. 5:**
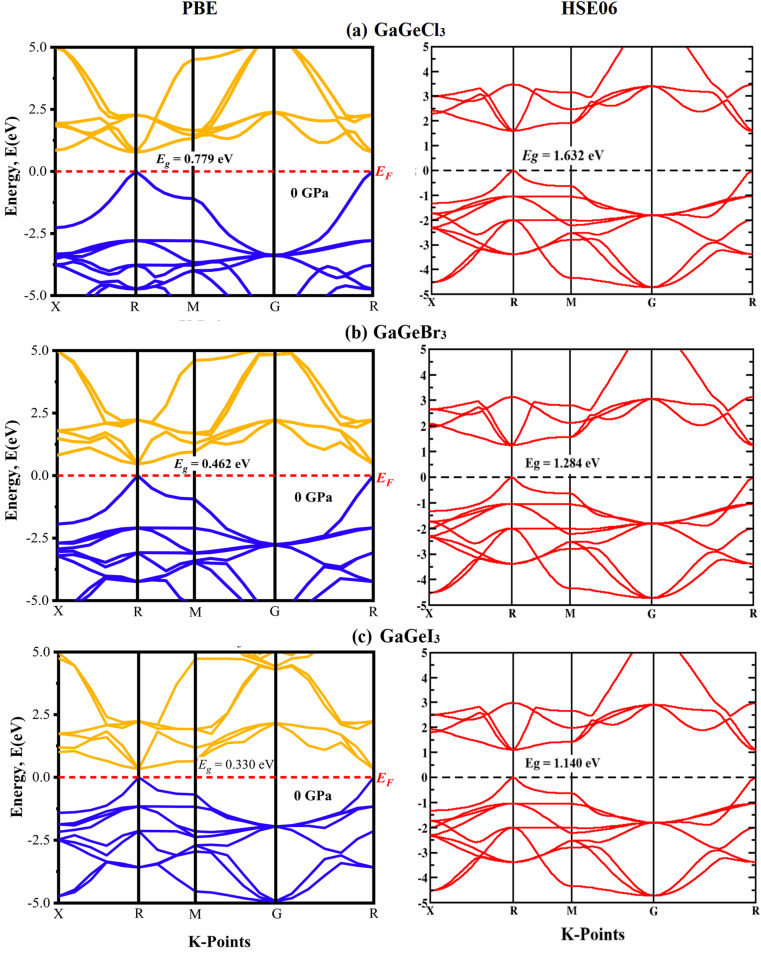
Calculated band dispersion diagram of (a) GaGeCl_3_ (b) GaGeBr_3_ and (c) GaGeI_3_ calculated by PBE and HSE06 functional.

**Fig. 6:**
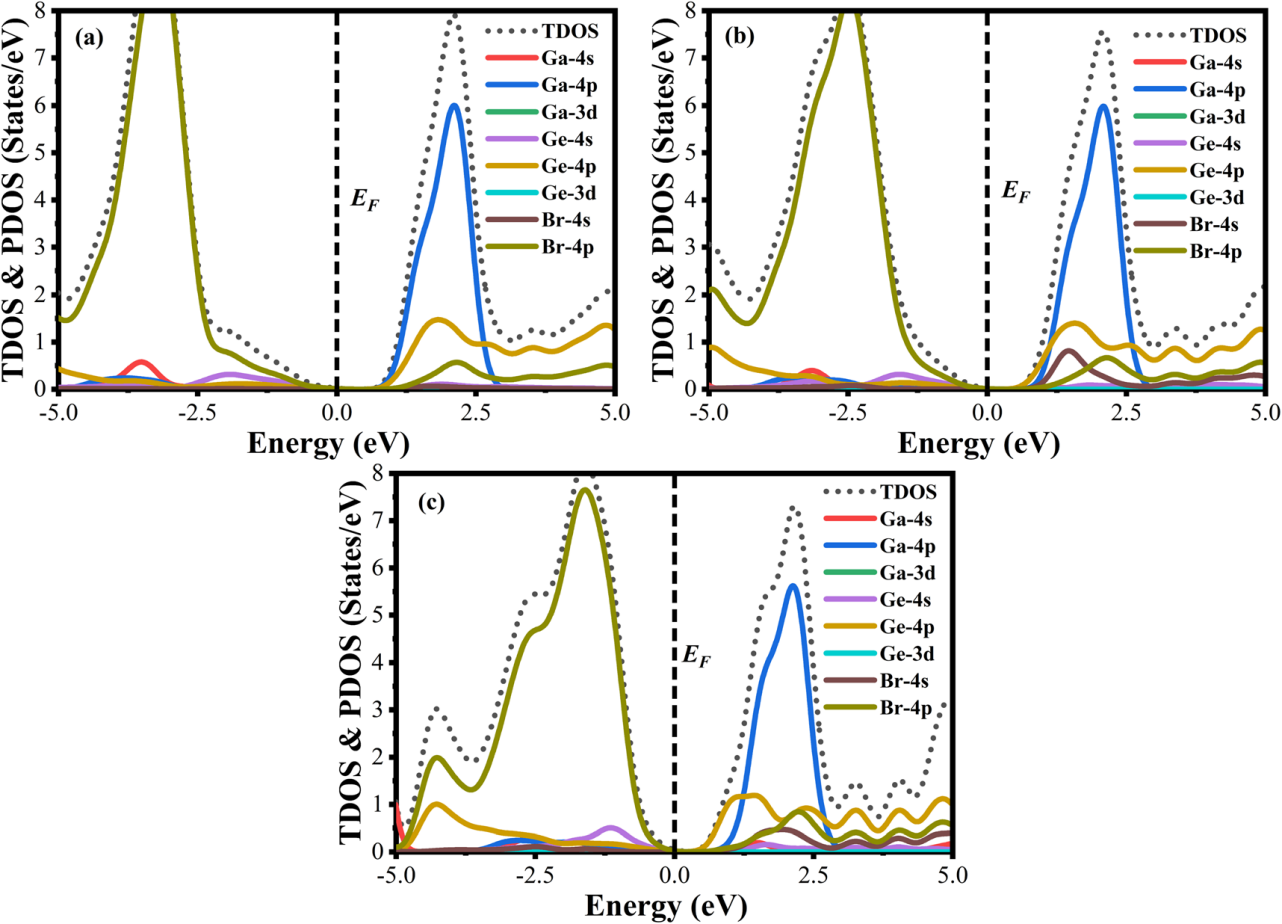
Total density of states (TDOS) and partial density of states (PDOS) of (a) GaGeCl_3_, (b) GaGeBr_3_, and (c) GaGeI_3_.

A material's band structure and density of states (DOS) have to be studied to assess its electrical nature. The electronic band dispersion profile of GaGeX_3_ (X = Cl, Br, and I) is therefore predicted using the PBE and HSE06 functionals, and it is depicted in Fig. 5 as well as listed in Table 1. Since GaGeX_3_ (X = Cl, Br, and I) lacks theoretical and experimental data, we have only compared the data among our investigated materials. The Fermi level in the illustration is marked by the red dashed line at 0 eV (*E*_F_). In addition, the valence band (VB) and conduction band (CB) are indicated by the blue lines below the Fermi level and the yellow lines above it, respectively. The energy dispersion profile elucidates that studied all compounds show a direct bandgap at the R–R point. GaGeCl_3_, GaGeBr_3_, and GaGeI_3_ have band gaps of 0.779 eV, 0.462 eV, and 0.330 eV, respectively, when calculated with the PBE functional, and 1.632 eV, 1.284 eV, and 1.140 eV, respectively, when calculated with the HSE06 functional. When Cl is substituted with halogen X (Br, and I), the conduction band is migrated toward the Fermi level and eventually energy gap is reduced. Reduced energy gap results from increased free electron intensity in the VB and CB, which is caused by the replacement of smaller halogens with bigger ones. Therefore, adjusting the halogen (X) concentration in GaGeX_3_ enables a tunable bandgap, a crucial feature for applications involving solar cells. To comprehensively assess the electronic properties, we computed and illustrated the density of states (DOS) for GaGeX_3_ (X = Cl, Br, and I) in Fig. 6. In this figure. The Fermi level is marked by the vertical dashed black line at 0 eV. To comprehend a material's semiconducting nature, the TDOS is essential. The figure confirms that all three of these materials are semiconductors under normal conditions since the TDOS of each material exhibits zero value close to the Fermi threshold. However, the PDOS is extremely important for understanding the contributions of the various atomic states in the band structure. Fig. 6 elicits that the VB of GaGeCl_3_ is mostly composed of Cl-3p orbitals, with little contribution from Ga-4s and Ge-4p orbitals, while the CB is primarily composed of Ga-4p and Ge-4p orbitals. As the bigger halogens X (Br and I) replace Cl, the energy gap is reduced as both the VB and CB peaks approach the Fermi level. In this instance, the VB comes primarily from Br-4p and I-5p, with a little Ge-4p contribution. Ga-4p is the most significant contributor to the formation of the CB, with minor contributions coming from Ge-4p, Br-4p/I-5p, and F–Br-4s/I-5s states. Here, the main responsibility for lowering the band gap *E*_g_ for all components lies in Ge-4p orbital. The band gap in GaGeX_3_ is a result of the hybridization of the orbitals of the atoms in the crystal structure. The origin of the band gap mainly come from hybridization of Cl-3p and Ga-4p, Ge-4p for GaGeCl_3_, Br-4p and Ge-4p for GaGeBr_3_, I-5p and Ge-4p for GaGeCl_3_ The band gap can be tuned by changing the halogen atom, as different halogens have different electronegativities and bond lengths, which affect the orbital overlap and the crystal field splitting.

#### 3.3.1 Electron localization funtion (ELF)

**Fig. 7:**
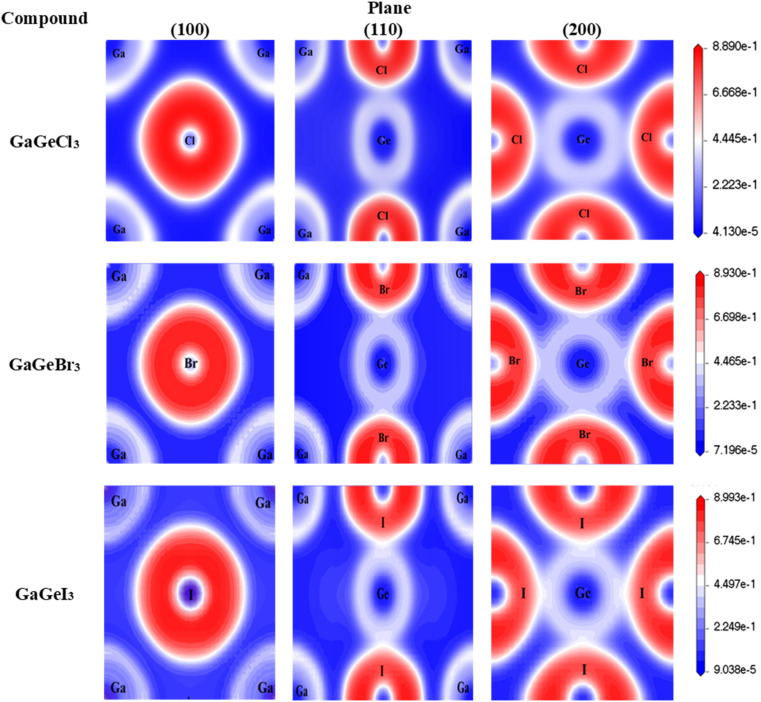
Mapping of Electron Localization Function (ELF) of GaGeX_3_ (X = CL, Br, and I) along (100), (110), and (200) planes.

ELF is based on the division of space into areas where electrons are concentrated. Alternatively, electrons are represented as a smooth charge density in conventional techniques. When it comes to systems with complicated bonding circumstances or significant electron correlation, ELF is relevant since it captures the localized character of electron density. Then, to investigate the chemical bonding characteristics of GaGeX_3_ (X = Cl, Br, and I), we have drawn their ELF maps in Fig. 7. We observed that halogen atoms such as Cl, Br, and I accumulate electrons (white red region) in the plane of (100), but Ga and Ge deplete electrons (white color region) in all situations.^57^ Moreover, the (100) plane displays spherical electron or charge distribution for the Cl–Ga, Br–Ga, and I–Ga atoms also no overlapping occurs between atoms which indicates antibonding states. This antibonding strength is higher for I compared to Cl and Br because I accumulates more electrons or charges and Ga depletes more electrons or charges in the case of Ga–I bonding. Then, the (110) plane represents the Ge-halogen and Ga–Ge bonding. In the case of Ga–Ge bonding, both the Ge and Ga atoms deplete electrons but they do not overlap between them. The depletion of electrons from the Ge atom is higher than the Ga atom and they possess antibonding. For a better understanding of the Ge-halogen bonding, we calculated ELF maps in the (200) plane. We see in Fig. 7 for the (200) plane that Ge atoms deplete electrons and create covalent bond with halogen atoms such as Cl, Br, and I. Also, we observe that Ge-halogen atoms overlap between them as well as an elliptical shape of electron distribution which indicates covalent bonding. Here, Ge and I atoms exhibit more ellipticity than Ge–Cl and Ge–Br atoms. Therfore, the bonding between Ge and I atoms is stronger compared to others.

### 3.4 Optical properties

Because of their exceptional optical properties, metal halide perovskites have attracted a lot of attention from scientists, especially in the field of photovoltaic cells and optoelectronic devices. The assessment of materials' performance in solar cells and optoelectronic devices is mostly dependent on critical factors, one of which is their optical properties. This section addresses the optical characteristics of GaGeX_3_ (X = Cl, Br, I), including the extinction coefficient, conductivity, reflectivity, real and imaginary portions of the dielectric function, absorption coefficient, and refractive index for different applications.

Of all the optical characteristics, the dielectric function is the most important one for a given material. Also, the dielectric function strongly connected with electronic band structure since contributions of the optical transitions to the dielectric function involve electron movements across different energy bands. For example, if the incoming photon energy matches the band gap energy, photon can excite electron from the VB to the CB.^58^ It is necessary to determine the dielectric function first to examine other optical characteristics. The dielectric function is commonly expressed as *ε*(*ω*) = *ε*_1_(*ω*) + *ε*_2_(*ω*), where *ε*_1_(*ω*), and *ε*_2_(*ω*) stand for the dielectric function's real and imaginary components, respectively. The Kramer–Kronig connection is used to determine the real component of the dielectric function, which has the following expression:^59^
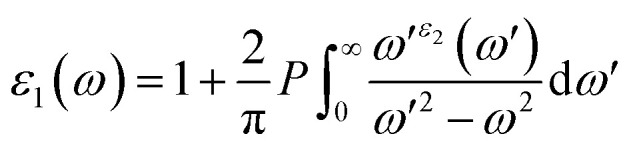


**Fig. 8:**
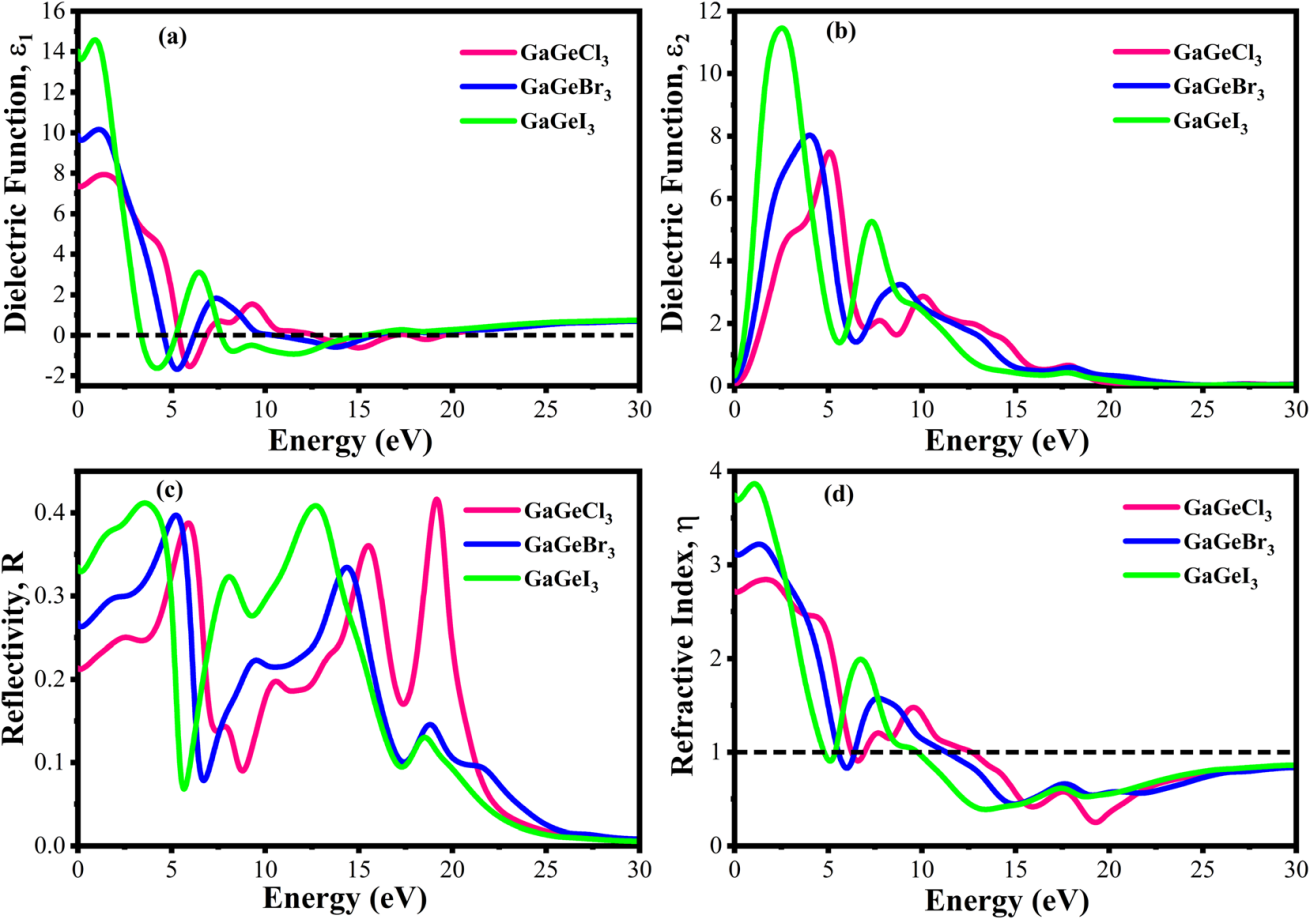
Calculated dielectric function (a) real part, (b) imaginary part, (c) reflectivity, and (d) refractive index of GaGeX_3_ (X = Cl ,Br, and I).

The value of *ε*_1_(*ω*) at 0 eV is referred to as the static dielectric function. It is an important metric for optoelectronic device efficiency. A material's low *E*_b_ (exciton binding energy) and decreased charge carrier recombination rate are indicated by a high static dielectric constant in that material. The real part of dielectric function of GaGeX_3_ (X = Cl, Br, and I) is shown in Fig. 8(a). The static dielectric constant of GaGeCl_3_ is 7.34, as can be shown in Fig. 8(a). On the other hand, the static dielectric constant value steadily rises when Cl is substituted by Br, or I. In particular, 9.86, and 14 are the static dielectric constants for NaGeBr_3_, and NaGeI_3_, respectively. Among the three compounds, GaGeI_3_ has the largest static dielectric constant value, which lowers the *E*_b_ and the rate of charge carrier recombination. For this reason, GaGeI_3_ performs better in solar cell applications in the photovoltaic industry. Fig. 8(a) also shows that GaGeCl_3_, GaGeBr_3_, and GaGeI_3_ exhibits negative value in the energy range of 12.6–19.7 eV, 10.4–15.9 eV, and 7.65–15.2 eV respectively, where they behave like metallic material and exhibit high reflectivity.

The real transition between the occupied and unoccupied electronic states may be used to calculate the imaginary dielectric constant (*ε*_2_), and it can be expressed as:
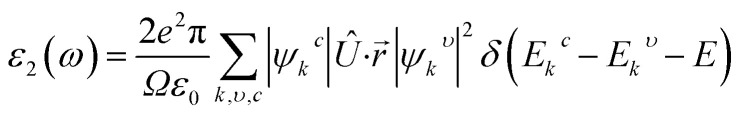


The *ε*_2_(*ω*) is a crucial parameter, as the absorption characteristics of a material are contingent upon it. It is significantly influenced by the band structure of the material. Fig. 8(b) displays the fluctuations of *ε*_2_(*ω*) in the energy range of 0 eV and 3 eV. It is clear from Fig. 8(b) that GaGeCl_3_ exhibits two prominent peaks, the largest of which is at 5.13 eV and the other at 10.1 eV. On the other hand, the peaks move into lower energy areas and become more intense when a more electronegative halogen X (Br and I) replaces Cl. In particular, for GaGeBr_3_ and GaGeI_3_, the largest peaks are seen at 4.01 eV and 2.61 eV, respectively. The illustration is further evident that the dielectric function spectra edge shifts towards the lower energy region from Cl to I, reaffirming that the band gap diminishes with increasing electronegativity of halogen X (Cl, Br, and I).

The optical characteristic of reflectance plays a crucial role in photovoltaic applications. An increased reflection in the visible spectrum adversely influences solar efficiency. The energy band gap and reflectivity are related properties that depend on how light interacts with a substance. Metals, for instance, exhibit high reflectivity because their electrons can readily absorb and re-emit light due to their zero band gap. Since insulators have wide band gaps and electrons find it difficult to move to higher energy levels, they have low reflectivity.^58^Fig. 8(c) demonstrates the reflectivity (*R*) spectrum of GaGeX_3_(X = Cl, Br, and I). As can be seen in Fig. 8(c), GaGeCl_3_ exhibits a very low reflectance of around 0.22 at 0 eV. However, the reflectance rises to 0.27 and 0.34 for GaGeBr_3_ and GaGeI_3_, respectively, when Cl is replaced with Br and I. Moreover, Fig. 8(c) shows that GaGeX_3_ (X = Cl, Br, and I) has higher reflectivity in the visible range, and GaGeI_3_ has the greatest level. The increased visual reflectance of GaGeI_3_ reduces its usefulness as a solar material. Therefore, more investigation is needed to reduce the reflectivity of GaGeI_3_ and improve its photovoltaic efficiency. Furthermore, GaGeX_3_ (X = Cl, Br, and I) has favorable reflectivity in the UV range, as shown in Fig. 8(c), indicating their possible application as coating materials to reduce solar heating in the UV range. Alongside this, GaGeI_3_ is predicted as a good material for applications involving UV shielding due to its wider reflectance spectrum in the UV area.

Fig. 8(d) shows the spectrum of refractive index. Notably, a gradual decline with increasing photon energy is demonstrated in Fig. 8(d), and the largest peak is recorded at 0 eV. Furthermore, the static refractive index of GaGeCl_3_ is 2.72, which is shown in Fig. 8(d). The trend shows a gradual rise from Cl to I, with GaGeBr_3_ and GaGeI_3_ reaching values of 3.11 and 3.7, respectively. It is clear by looking at CsCdX_3_ (X = Cl, Br, and I) that these compounds have the highest refractive index in the infrared area and the lowest in the ultraviolet. When refractive index goes below unity, the group velocity of the incident photon becomes higher than light velocity, and this characteristic is familiar as superluminal. Among the compounds, CsCdI_3_ has the greatest refractive index at 0 eV, which makes it suitable for use in waveguide applications.^60^

**Fig. 9:**
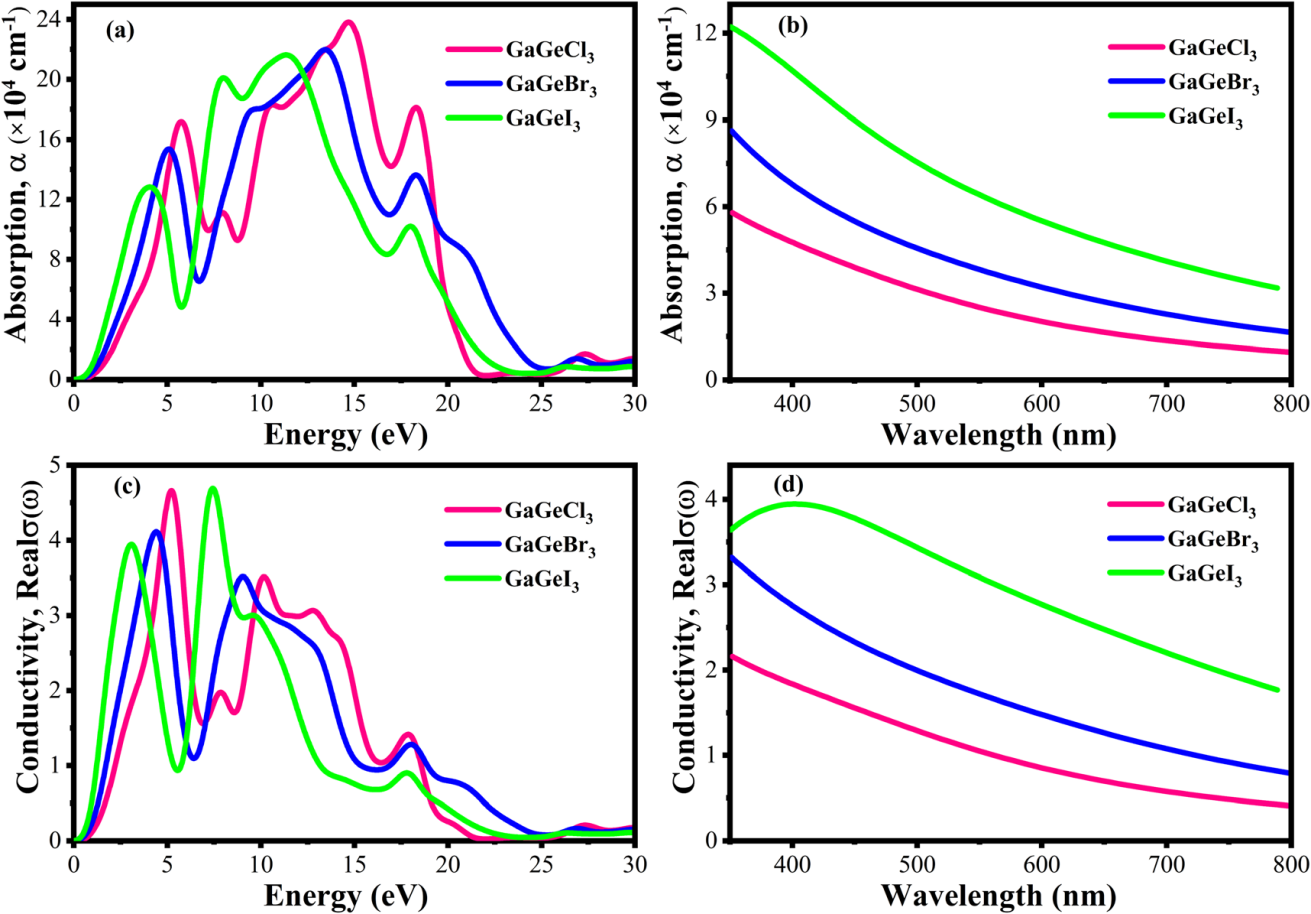
(a) Absorption coefficient *vs.* energy (b) absorption coefficient *vs.* wavelength (c) conductivity *vs.* energy, and (d) conductivity *vs.* wavelength, of GaGeX_3_ (X = Cl, Br, and I).

The efficiency of solar cells and other optoelectronic devices is significantly influenced by the absorption coefficient (*α*), as it conveys important details regarding the absorbing capability of a certain material. It is one of the numerous optical characteristics whose performance is significantly affected by it. For effective absorption, the incoming photons energy must meet or greater than the band gap energy. Besides, the probability of absorption is higher when there is a large density of states present at any given energy level.^58^Fig. 9(a and b) shows the fluctuation of *α* as a function of photon energy. Four prominent peaks within the energy range of 0–30 eV are displayed for each compound. In Fig. 9(a), it is observed that GaGeCl_3_ reaches its maximum peak at 14.7 eV. Conversely, when Cl is substituted with Br and I, a redshift occurs, and the highest peaks are observed at 13.5 eV and 11.4 eV, respectively, for GaGeBr_3_ and GaGeI_3_. Additionally, GaGeI_3_ is the most absorption-efficient material among the three materials in the visible region, as seen in Fig. 9(b). It suggests that GaGeI_3_ is a better candidate than the other two for use as a solar cell material. Overall, all compounds can be used in multijunction solar cell due to their high absorbtion coefficient.

A material's photoconductivity (*σ*) is used to measure how many photons are able to pass through it. Also, conductivity is directly related with electronic band structure such as energy gap between VB and CB, presence of free charge carrier of these bands. Therefore, large band gap shows lower conductivity and overlapping or tightly binded band shows higher conductivity.^58^Fig. 9(c and d) shows the conductivity spectra of GaGeX_3_ (X = Cl, Br, and I), which are comparable to the absorption spectrum of GaGeX_3_(X = Cl, Br, and I), as they are obtained from the absorption spectra. Fig. 9(d) illustrates that GaGeI_3_ has a higher photoconductivity in the visible spectrum at almost 3.95 fs^−1^, while GaGeBr_3_ and GaGeCl_3_ have 3.44 fs^−1^ and 2.21 fs^−1^, respectively. The conductivity spectra of GaGeX_3_ (X = Cl, Br, and I) with two notable peaks in the 0–30 eV energy region are shown in Fig. 9(c). GaGeCl_3_ exhibits these peaks at 5–5.5 eV and 9.5–10.5 eV, having the largest peak at 5.26 eV. A redshift happens when the higher electronegativity halogens X (Br and I) are substituted for Cl. Therefore, GaGeBr_3_ shows prominent peaks at 4–5 eV and 8.5–9.5 eV respectively, with the highest peak occurring at 4.47 eV. Conversely, GaGeI_3_ has two major peaks at 2.5–3.5 eV and 7–8 eV respectively, with the most intense peak taking place at 7.44 eV.

**Fig. 10:**
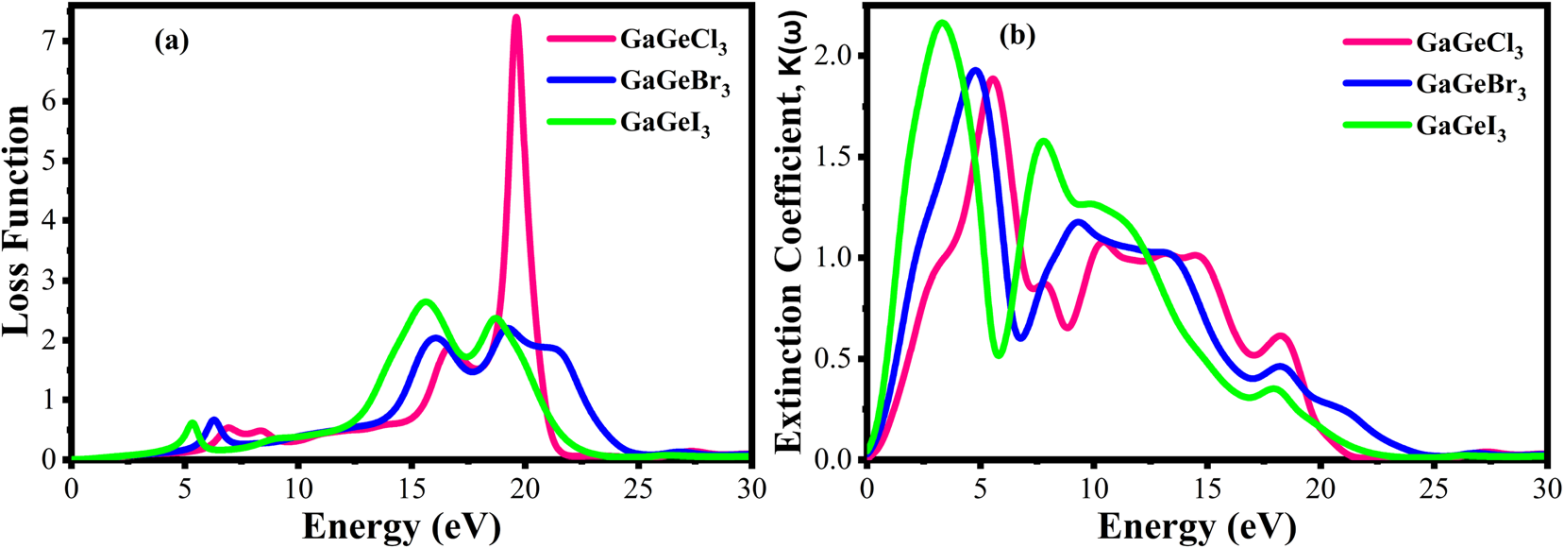
Calculated (a) loss function *vs.* energy, and (b) extinction coefficient *vs.* energy of GaGeX3 (X = Cl, Br, and I).

Fig. 10(a) displays the loss functions of our studied compounds. Energy regions in an atom where electrons are usually not restricted to their lattice locations and exhibit a plasma frequency when illuminated are described by the loss function of energy. It is evident from Fig. 8(c) and 10(a) that the reflectivity of GaGeX_3_ (X = Cl, Br, and I) abruptly decreases at the locations where the loss function peaks. Additionally, Fig. 10(b) displays the extinction coefficient *K*(*ω*) of GaGeX_3_ (X = Cl, Br, and I). The illustrations (Fig. 8(b), and 10(b)) show that the extinction coefficient spectrum and *ε*_2_(*ω*) have similar patterns. In particular, GaGeCl_3_ has the lowest value for *K*(*ω*), and this value rises when Br and I are substituted for Cl. Additionally, when bigger halogens, X (Br and I), replace Cl, the peaks in the spectrum move towards the lower energy region.

## 4. Conclusion

The current study used the DFT-based FP-LAPW approach to thoroughly examine the structural, elastic, and optical characteristics of cubic lead-free halide perovskites GaGeX_3_ (Cl, Br, and I). According to formation energy, Born stability criteria, and phonon analysis, all of our interested compounds are stable. GaGeI_3_ has the greatest cell volume and lattice parameter among these three compounds, whereas GaGeCl_3_ has the lowest. Moreover, GaGeX_3_ (X = Cl, Br, and I) are ductile, and GaGeBr_3_ shows the highest level of ductility, which is supported by Cauchy pressure, Poisson's and Pugh's ratios. In addition to higher ductility, GaGeBr_3_ shows superior machinability. Furthermore, the band structure reveals that all three compounds are direct bandgaps along the R–R direction, and GaGeCl_3_ shows the highest bandgap among them. However, by simply adjusting the halogen levels, these material's band gaps can be modified, which makes them appropriate for multijunction solar cell applications. Compared to the other compounds, GaGeI_3_ has greater light absorption, stronger conductivity in visible areas, a higher dielectric constant, which denotes a lower exciton binding energy, *E*_b_, and a lower recombination rate. The comprehensive optical, electronic, and mechanical properties of these materials render them suitable for photovoltaic applications, marking a significant contribution to the field of renewable energy technologies. The study not only advances our understanding of halide perovskites but also demonstrates their practical potential in the development of next-generation electronic devices.

## Data availability

The datasets and computer codes are available upon request from the authors.

## Author contributions

Md. Amran Sarker: investigation, methodology, data curation and analysis and interpretation, writing the original draft. Md Mehedi Hasan: investigation, methodology, data curation, graph ploting. Md. Al Momin: investigation, methodology, writing the original draft. Ahmad Irfan: review & editing. Md. Rasidul Islam: bandgap calculation by HSE06 functional, phonon analysis. Ahmed Sharif: resources allocation, Supervision, writing – review & editing.

## Conflicts of interest

The authors declare no conflict of interest.

## Supplementary Material
